# Feeding soy protein isolate and oils rich in omega-3 polyunsaturated fatty acids affected mineral balance, but not bone in a rat model of autosomal recessive polycystic kidney disease

**DOI:** 10.1186/s12882-015-0005-9

**Published:** 2015-02-10

**Authors:** Kaitlin H Maditz, Brenda J Smith, Matthew Miller, Chris Oldaker, Janet C Tou

**Affiliations:** Division of Animal and Nutritional Sciences, West Virginia University, 1038 Agricultural Sciences Bldg, P.O. Box 6108, Evansdale Campus, Morgantown, West Virginia 26505 USA; Department of Nutritional Sciences, Oklahoma State University, Stillwater, OK 74078 USA

**Keywords:** Omega-3 fatty acids, Soy protein, Mineral balance, Long bones, Polycystic kidney disease

## Abstract

**Background:**

Polycystic kidney disease (PKD), a genetic disorder characterized by multiple cysts and renal failure at an early age. In children, kidney disease is often accompanied by disordered mineral metabolism, failure to achieve peak bone mass, and reduced adult height. Optimizing bone health during the growth stage may preserve against bone loss associated with early renal dysfunction in PKD. Dietary soy protein and omega-3 polyunsaturated fatty acid (n-3 PUFA) have been reported to ameliorate PKD and to promote bone health. The study objective was to determine the bone effects of feeding soy protein and/or n-3 PUFAs in a rat model of PKD.

**Methods:**

Weanling female PCK rats (n = 12/group) were randomly assigned to casein + corn oil (Casein + CO), casein + soybean oil (Casein + SO), soy protein isolate + soybean oil (SPI + SO) or soy protein isolate + 1:1 soybean oil:salmon oil blend (SPI + SB) for 12 weeks.

**Results:**

Rats fed SPI + SO diet had shorter (*P* = 0.001) femur length than casein-fed rats. Rats fed SPI + SO and SPI + SB diet had higher (*P* = 0.04) calcium (Ca) and phosphorus (P) retention. However, there were no significant differences in femur and tibial Ca, P or bone mass between diet groups. There were also no significant difference in bone microarchitecture measured by micro-computed tomography or bone strength determined by three-point bending test between diet groups.

**Conclusions:**

Early diet management of PKD using SPI and/or n-3 PUFAs influenced bone longitudinal growth and mineral balance, but neither worsened nor enhanced bone mineralization, microarchitecture or strength.

## Background

Kidney disease in children is often accompanied by disordered mineral metabolism, decreased adult height, and failure to achieve peak bone mass (PBM) [1,2]. Epidemiological studies demonstrated greater incidence of bone fracture in end stage renal disease patients [3]. Worldwide, the third most common cause of renal failure is polycystic kidney disease which is characterized by multiple cysts, massive enlargement of the kidneys, and loss of renal function [4]. In autosomal dominant polycystic kidney disease (ADPKD), the most prevalent inherited form of polycystic kidney disease, individuals are asymptomatic until the third or fifth decades of life [5]. In the rarer autosomal recessive polycystic kidney disease (ARPKD), disease onset occurs early with more than 50% of affected children progressing to renal failure in the first decade of life [5,6]. Common strategies for the management of renal disease in childhood include maintaining a healthy body weight and maximizing PBM [7].

Soy protein isolate (SPI) and omega-3 polyunsaturated fatty acids (n-3 PUFAs) are dietary components reported in pre-clinical studies to ameliorate polycystic kidney disease progression and severity [7]. The PCK rat, an orthologous model of human ARPKD, has been used in pre-clinical testing of drug treatments [8]. To our knowledge no studies have investigated the effects of dietary SPI on PCK rats. The PCK rat is derived from a spontaneous mutation in Sprague–Dawley rats [9]. Growing female Sprague–Dawley rats fed SPI have been reported to have enhanced bone mineral content (BMC) and bone mineral density (BMD) compared to casein [10]. Some potential bone protective effects of SPI are the presence of estrogenic isoflavones and an amino acid profile associated with reduced acid load [11]. Diet acid load is a major determinant of calcium (Ca) excretion and in turn, bone mineral loss [11].

Different n-3 PUFA sources also affect bone. Growing female Sprague–Dawley rats fed fish oil rich in the long-chain n-3 PUFAs, eicosapentaenoic acid (EPA, 20:5n-3) and docosahexaeonic acid (DHA, 22:6n-3), increased long bone BMD and BMC compared to rats fed corn oil low in n-3 PUFAs [12]. Growing female Hans:SPRD-*cy* rats, a non-orthologous model of human ADPKD, fed ALA-rich flaxseed oil had higher whole body BMC and BMD compared to animals fed corn oil [13]. To our knowledge no studies have investigated whether dietary n-3 PUFAs enhances bone health in ARPKD.

Studies on the effects of ARPKD on bone are lacking because treatment priority is on slowing disease progression to renal failure and early mortality [7]. Some drug therapies in development to treat polycystic kidney disease have reported side effects that include bone loss [14]. Also, many drugs used in the treatment of osteoporosis are not approved for use in patients with moderate to advanced kidney diseases [3]. Early diet management by optimizing bone health during the growth stage can potentially minimize bone loss due to renal dysfunction at an early age in polycystic kidney disease patients. Therefore, the objective of this study was to determine whether feeding growing female PCK rats SPI and n-3 PUFA can affect mineral balance and optimize bone health. Female rats were used because they are reportedly more efficient at metabolizing dietary ALA to the bioactive n-3 PUFAs, EPA and DHA [15]. Female Hans:SPRD-*cy* rats fed diets supplemented with phytoestrogens had more beneficial effects on PKD progression than male rats [16]. Also, slower progression of PKD in females enable the subtle influence of diet to be detectable [7].

## Methods

### Animals and diets

All animal procedures were conducted in accordance with the Institute of Laboratory Animal for the Care and Use of Laboratory Animals Guidelines [17], and were approved by the Animal Care and Use Committee at West Virginia University. Growing (age 28 d) female PCK rats were purchased from Charles River Laboratories (Wilmington, MA). All rats were kept in a 21°C room with a 12 hour light/dark cycle throughout the 12 week study period. Following a 7- day acclimation period, animals were randomly assigned (n = 12 rats/group) to experimental diets. A power analysis conducted prior to the experiment showed that with 10 rats/group at an alpha = 0.05 there is 80% power to detect a difference in BMD and bone volume per unit of total volume (BV/TV) between the diet groups. An additional 2 rats/group was included due to possibility of morbidity or mortality due to disease severity in PCK rats.

All experimental diets were manufactured by Harlan Teklad (Madison, WI). Diet ingredients are provided in Table 1. The experimental diets were based on the American Institute of Nutrition-93G (AIN-93 G) standard rodent diet consisting of purified ingredients formulated to meet all the nutritional requirements of growing rats as defined by the National Research Council [18]. The AIN-93G diet is formulated with casein as the protein source (200 g/kg diet) and soybean oil as the lipid source (70 g/kg diet). In this study, experimental diets consisted of either casein or SPI as the protein source. SPI was generously provided by DuPont Nutrition and Health (St. Louis, MO). Both protein sources consisted of 87% crude protein. Caloric value was 4.3 kcal/g for SPI and 4.2 kcal/g for casein.

**Table 1:** **Experimental diet composition**

**Ingredient (g/kg diet)** ^**1**^	**Casein + CO**	**Casein + SO**	**SPI + SO**	**SPI + SB**
Casein	200.0	200.0	0.0	0.0
Soy protein isolate	0.0	0.0	200.0	200.0
L-Cystine	3.0	3.0	1.3	1.3
Corn Starch	397.5	397.5	397.5	397.5
Maltodextrin	132.0	132.0	132.0	132.0
Sucrose	100.0	100.0	100.0	100.0
Soybean Oil	70.0	0.0	70.0	35.0
Corn Oil	0.0	70.0	0.0	0.0
Salmon Oil	0.0	0.0	0.0	35.0
Cellulose	50.0	50.0	50.0	50.0
Mineral Mix^2^	35.0	35.0	35.0	35.0
Vitamin Mix^2^	10.0	10.0	10.0	10.0
Choline bitartrate	2.5	2.5	2.5	2.5
TBHQ, antioxidant	0.014	0.014	0.014	0.014
Calories	377	377	377	377
**Fatty acids (% total fatty acids)**				
*n-3 PUFAs*				
Alpha-linolenic acid (ALA, 18:3n-3)	0.59 ± 0.18c	7.69 ± 0.02^a^	7.68 ± 0.04^a^	4.85 ± 0.01^b^
Eicosapentaenoic acid (EPA, 20:5n-3)	ND	ND	ND	7.4 ± 0.03
Docosahexaenoic acid (DHA, 22:6n-3)	ND	ND	ND	3.9 ± 0.1
*n-6 PUFAs*				
Linoleic acid (LA, 18:2n-6)	52.93 ± 2.68^a^	55.78 ± 0.07^a^	56.09 ± 0.09^a^	35.19 ± 0.06^b^
Arachidonic acid (ARA, 20:4n-6)	1.10 ± 0.04^a^	0.16 ± 0.005^c^	0.21 ± 0.01^c^	0.41 ± 0.007^b^
n-3/n-6	0.01	0.14	0.14	0.44

^1^All ingredients are from Harlan Teklad except for SPI from DuPont Nutrition and Health (St. Louis, MO) and salmon oil from JEdwards International (Quincy, MA).

^2^Based on the AIN-93G vitamin and mineral mixes [36]. Different superscript letters a, b, c within the same rows indicate significant differences at *P* < 0.05 by one-way ANOVA followed by Tukey’s test. Abbreviations are *CO* corn oil, *SO* soybean oil, *SPI* soy protein isolate, *SB* 1:1 soybean oil:salmon oil blend, *TBHQ* tertiary butylhydroquinone, *ND* not detectable.

The lipid sources consisted of corn oil because it is high in n-6 PUFA and low in n-3 PUFA which is typical of Western diets [19]. Soybean oil was included because it contains both essential fatty acids, the n-6 PUFA, linoleic acid (LA, 18:2n-6) and the n-3 PUFA, ALA. A 1:1 ratio of soybean oil:salmon oil was included because it contains the n-3 PUFAs, ALA, EPA and DHA. Salmon oil was purchased from JEdwards International Inc (Quincy, MA). Caloric value was 9.2 kcal/g for corn oil, 9.1 kcal/g for soybean oil, and 9.3 kcal/g for the 1:1 soybean oil:salmon oil blend.

The experimental diets consisted of: 1) casein + corn oil (Casein + CO), 2) casein + soybean oil (Casein + SO), 3) soy protein isolate + soybean oil (SPI + SO) or 4) soy protein isolate + 1:1 soybean/salmon oil (SPI + SB). Caloric value was 3.77 Kcal/g and metabolizable energy was 16.1 ± 0.3 kJ/g for all diets. Using inductively coupled plasma spectrometry (ICP, model P400, Perkin Elmer, Shelton, CN), dietary Ca content was determined to be higher (*P =* 0.04) in the SPI + SO (9.7 ± 0.1 mg/g) and SPI + SB (10.4 ± 0.1 mg/g) compared to Casein + SO (8.8 ± 0.1 mg/g) and Casein + CO (8.2 ± 0.2 mg/g) diets. Dietary phosphorus (P) content was determined to be higher (*P =* 0.03) in the SPI + SO (7.3 ± 0.1 mg/g) and SPI + SB (7.2 ± 0.1 mg/g) compared to Casein + SO (6.1 ± 0.5 mg/g) and Casein + CO (6.5 ± 0.2 mg/g) diets.

All diets were kept at −20°C until fed. Rats were provided their assigned diet and deonized distilled water (ddH_2_O) to prevent mineral intake from sources other than the diet. Body weights were measured weekly throughout the 12 week study.

### Mineral balance

To prevent variability in food intake, rats were provided 15 ± 2 g of powdered diet daily. This amount has been reported to support growth based on body weights [20]. Food intake was measured daily and replaced with fresh diet. During the final week of the 12 weeks feeding study, rats were individually housed in metabolic cages to collect urine and feces. Final 7 day urine samples were collected, centrifuged at 1,500 g for 10 min at 4°C, filtered through Whatman no. 1 paper, and diluted 1:10 in ddH_2_O. Urinary Ca and P concentration were determined using ICP (model P400, Perkin Elmer, Shelton, CN).

Diet and fecal samples were freeze-dried for 48 h, ashed in a muffle furnace at 550°C for 24 h, and then acidified in 70% nitric acid. The acidified samples were neutralized in ddH_2_O, filtered through Whatman no. 1 paper, diluted 1:50 in ddH_2_O, and both diet and fecal Ca and P content determined by ICP.

Following euthanasia by CO_2_ inhalation, the chest cavity was opened and the aorta punctured to collect blood. Blood was centrifuged at 1,500 *g* for 10 min at 4°C to obtain serum. Serum samples were stored at −80°C until assayed. Serum parathyroid hormone (PTH) was determined by commercially available rat enzyme immunoassay (EIA) kit (RayBiotech Inc., GA). Absorbance was determined at 450 nm using a Spectramax Plus microplate reader (Molecular Devices, Sunnyvale, CA). Serum Ca and P were determined enzymatically using a commercially available Vet-16 rotor and quantified by a Hemagen Analyst automated spectrophotometer (Hemagen Diagnostics Inc., Columbia, MD).

### Bone morphometry

Following euthanization by CO_2_ asphyxiation, the right and left femurs and tibiae were collected (n = 12 rats/group). Bones were defleshed and morphometry measurements of medial lateral width, anterior posterior width, and length were determined using a vernier caliper (Bel-Art Products, Pequannock, NJ). Length was measured from the medial condyle to greater trochanter. Bones were dried at 110°C for 48 h then weighed using an analytical balance (Mettler Toledo, Columbus, OH). Morphometry measurements were averaged for bone pairs (i.e. right and left) after no bilateral differences were determined using paired t-test with significance level set at *P* < 0.05.

### Bone densitometry, Ash, Ca, and P

Dual energy X-ray absorptiometry (DXA) scans (Hologic QDR 4500-A Elite) were performed on the left bones. Tibial and femoral specimens were placed in Millipore water. Bone mineral area (BMA), BMC, BMD were evaluated from all scans using the Regional High Resolution software package designed for studying isolated bone specimens (Hologic Waltham, MA).

Following DXA, bones were ashed in a muffle furnace (model CP18210, Thermolyne, Dubuque, IA) at 600°C for 24 h then weighed. Bone ash was dissolved in 2 mL of 70% nitric acid. Acidified samples were filtered through Whatman no. 1 paper and diluted (1:500 v/v) to volume with ddH_2_O. Bone Ca and P content were measured using ICP.

### Bone microarchitectural analyses

Trabecular and cortical bone architecture was determined in the left bones using micro-computed tomography (μCT) (MicroCT40, SCANCO Medical, Switzerland). Cortical bone architecture was evaluated on a 512 μm region of the femur and tibia mid-diaphysis. Cortical indices included cortical thickness, cortical area, medullary area, and porosity. The trabecular bone within the distal femur metaphysis and proximal tibial metaphysis were scanned and 200 images (~16 μm/slice or 3.2 mm) were analyzed with semi-automatically drawn contours beginning 400 μm from the growth plate including only secondary spongiosa within the volume of interest. The volume of interest was assessed for structural parameters included trabecular BV/TV, trabecular number (TbN), trabecular thickness (TbTH), trabecular separation (TbSp), trabecular connectivity (Conn), and structure model index (SMI). All scans were performed utilizing a 1024 × 1024 matrix resulting in an isotropic voxel resolution of 22 μm^3^. An integration time of 70 milliseconds per projection was used with a rotational step of 0.36° resulting in total acquisition time of approximately ~150 min/sample.

### Bone biomechanical strength

Bone strength indices were assessed using a TA.XT2i Texture Analyzer (Texture Technologies, Scarsdale, NY) outfitted with a three-point bending apparatus. Femora and tibiae were placed on supports and force applied on the medial surface of the bones by lowering a centrally placed blade (1 mm width) at a constant crosshead speed (0.1 mm/sec) until the bone was broken. The load cell was 250 kg. The load-deflection data were collected by a PC interfaced with the TA.XT2i. Sample test distance was set at 10 mm with a signal collection rate of 100 points per sec. A force-displacement curve generated from the biomechanical test was used to determine bone strength measurements. Peak force is the highest load obtained before bone fracture occurs. Ultimate stiffness is the slope of the curve responding to bone stiffness [21]. Ultimate bending stress is a normalized calculated force that takes into account the size of the bone and Young’s Modulus is a normalized calculated bone stiffness that takes into account bone size [22].

### Bone turnover markers

Serum osteocalcin concentration was determined using a commercially available rat specific EIA (Biomedical Technologies, Stoughton, MA). Urinary deoxypyridinoline (DPD) concentration was determined by commercially available EIA kit (Quidel Corporation, CA). Serum osteocalcin was measured at 450 nm and urinary DPD was measured at 405 nm using a Spectramax Plus microplate reader.

### Statistical analysis

Results are expressed as means ± standard error of the mean (SEM). One way analysis of variance (ANOVA) was used to determine differences between treatment groups. Post-hoc multiple comparisons were performed using Tukey’s test (parametric) or Wilcoxon rank test (non-parametric). Differences were considered significant at *P* < 0.05. All statistical analyses were performed using SigmaStat 3.1 statistical software program (Systat Software Inc., San Jose, CA).

## Results

### Body Weight and Bone Morphometry

**Figure 1:**
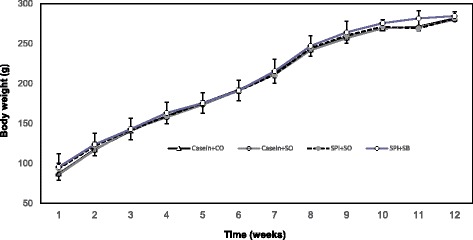
**The effect of feeding growing PCK female rats soy protein isolate and/or omega-3 polyunsaturated fatty acids on body weight during the 12 week feeding study.** Values are the mean ± SEM of n = 12 rats/diet group. Abbreviations are casein + corn oil (Casein + CO), casein + soybean oil (Casein + SO), soy protein isolate + soybean oil (SPI + SO) or soy protein isolate + 1:1 soybean/salmon oil (SPI + SB).

Growing female PCK rats fed different dietary treatments for 12 weeks had similar body weights (Figure 1). Femur length was shorter (*P* = 0.001) in PCK rats fed SPI + SO diet compared to rats fed Casein + CO and Casein + SO diets (Table 2). There were no significant differences in femur width or dry weight between any of the diet groups. There were no differences in tibia length, width, and dry weight.

**Table 2:** **Bone morphometry in growing female PCK rats fed different diets**

Measurement*	**Casein + CO**	**Casein + SO**	**SPI + SO**	**SPI + SB**	***P*** **-value**
**Femur**					
Length (mm)	31.01 ± 0.13^a^	31.23 ± 0.15^a^	30.17 ± 0.28^b^	30.74 ± 0.14^ab^	0.001
Medial Lateral width (mm)	3.77 ± 0.02	3.75 ± 0.03	3.78 ± 0.02	3.79 ± 0.03	0.78
Anterior posterior width (mm)	2.78 ± 0.03	2.77 ± 0.03	2.75 ± 0.03	2.84 ± 0.03	0.22
Dry weight (g)	0.69 ± 0.04	0.70 ± 0.04	0.68 ± 0.46	0.68 ± 0.04	0.98
**Tibia**					
Length (mm)	36.24 ± 0.15	36.45 ± 0.07	36.20 ± 0.07	36.17 ± 0.11	0.25
Medial Lateral width (mm)	2.24 ± 0.02	2.29 ± 0.02	2.24 ± 0.02	2.29 ± 0.02	0.18
Anterior posterior width (mm)	2.68 ± 0.02	2.67 ± 0.02	2.67 ± 0.02	2.66 ± 0.02	0.88
Dry weight (g)	0.47 ± 0.02	0.48 ± 0.02	0.46 ± 0.02	0.47 ± 0.02	0.95

*Values are the means ± SEM of n = 12 rats/group. Different superscript letters a, b within the same rows indicate significant differences at *P* < 0.05 by one-way ANOVA followed by Tukey’s test. Abbreviations are *CO* corn oil, *SO* soybean oil, *SPI* soy protein isolate, *SB* 1:1 soybean oil:salmon oil blend.

### Mineral intake and balance

Urinary output was higher (*P* = 0.01) in rats fed SPI diets compared to rats fed casein diets, but there were no differences (*P* = 0.79) in water intake. Ca intake was significantly higher in rats fed SPI diets compared to rats fed casein diets. However, urinary Ca excretion was lower (*P* = 0.02) in rats fed SPI + SO diets compared to rats fed casein diets. Rats fed SPI diets had higher (*P* = 0.04) Ca retention than rats fed casein diets. There was no significant differences in fecal Ca excretion or Ca apparent absorption between diet groups (Table 3).

**Table 3:** **Calcium and phosphorus balance in growing female PCK rats fed different diets**

Measurement*	**Casein + CO**	**Casein + SO**	**SPI + SO**	**SPI + SB**	***P*** **-value**
Water intake (mL/d)	14.4 ± 9.4	15.2 ± 13.7	16.3 ± 11.5	16.5 ± 12.3	0.79
Urinary Output (mL/d)	2.1 ± 0.2b	2.3 ± 0.2b	3.8 ± 0.3a	3.5 ± 0.3a	0.01
Fecal Output (mg/d)	5.2 ± 0.3	5.0 ± 0.3	5.5 ± 0.3	5.7 ± 0.2	0.21
**Calcium**					
Ca Intake (mg/d)	131.3 ± 2.4b	123.0 ± 2.2b	144.7 ± 2.1a	156.6 ± 2.4a	<0.001
Urinary Ca Excretion (mg/mL)^1^	1.9 ± 0.3a	1.6 ± 0.5a	0.5 ± 0.07b	1.3 ± 0.3ab	0.02
Fecal Ca Excretion (mg/d)	29.9 ± 2.3	27.2 ± 2.3	26.0 ± 2.2	29.0 ± 2.7	0.65
Ca Apparent Absorption (%)^2^	77.2 ± 1.7	77.9 ± 1.9	82.0 ± 1.5	81.5 ± 1.7	0.12
Ca Retention (mg/d)^3^	75.7 ± 1.7b	76.6 ± 1.8b	81.7 ± 1.5a	80.6 ± 1.7a	0.04
**Phosphorus**					
P Intake (g)	91.8 ± 1.0b	97.0 ± 2.1b	109.8 ± 2.0a	108.6 ± 2.3a	<0.001
Urinary P Excretion (mg/mL)^4^	7.9 ± 1.0a	6.8 ± 1.3a	3.4 ± 0.6b	2.3 ± 0.6b	0.01
Fecal P Excretion (mg/d)	26.1 ± 2.6	28.4 ± 1.3	28.3 ± 2.5	26.3 ± 2.7	0.85
P Apparent Absorption (%)^5^	71.6 ± 2.9	70.7 ± 1.4	74.2 ± 2.3	75.7 ± 2.5	0.42
P Retention (mg/d)^6^	63.0 ± 3.1b	63.7 ± 1.8b	71.1 ± 2.5a	73.6 ± 2.3a	0.04
Serum parathyroid hormone (pg/mL)	36.2 ± 4.1	37.2 ± 4.4	39.9 ± 5.2	43.6 ± 5.9	0.74
Serum Ca (mg/mL)	0.12 ± 0.004	0.12 ± 0.001	0.11 ± 0.004	0.11 ± 0.004	0.17
Serum P (mg/mL)	0.12 ± 0.006	0.13 ± 0.007	0.11 ± 0.006	0.12 ± 0.005	0.32

^*^Values are expressed as the mean _±_ SEM of n = 12 rats/group. Different letters a, b within the same column indicate significant differences at *P <* 0.05 by one-way ANOVA followed by Tukey’s test. Abbreviations are *SO* soybean oil, *SPI* soy protein isolate, *SB* 1:1 soybean oil:salmon oil blend, *Ca* calcium; *P* phosphorus.

^1^Urinary calcium excretion was calculated as urinary Ca concentration/urine volume.

^2^Calcium apparent absorption was calculated as [(Ca intake – fecal Ca excretion)/Ca intake] x 100.

^3^Calcium retention was determined by calculating [Ca intake – (fecal Ca excretion + urinary Ca excretion)].

^4^Urinary phosphorus excretion was calculated as urinary P concentration/urine volume.

^5^Phosphorus apparent absorption was calculated as [(P intake – fecal P excretion)/P intake] x 100.

^6^Phosphours retention was determined by calculating [P intake – (fecal P excretion + urinary P excretion)].

P intake was significantly higher in rats fed SPI diets compared to rats fed casein diets. However, urinary P excretion was lower (*P* = 0.01) in rats fed SPI diets compared to rats fed casein diets. Rats fed SPI diets had higher (*P* = 0.04) P retention than rats fed casein diets. There were no significant differences in fecal P excretion or P apparent absorption between diet groups (Table 3). There were no significant differences in serum PTH, Ca or P concentrations among the dietary groups.

### Bone mineral analyses

Shown in Table 4, there were no significant differences in femur or tibial Ca and P between diet groups. Additionally, there were no significant differences in long bone ash, BMA, BMC, and BMD between rats fed Casein + CO, Casein + SO, SPI + SO, and SPI + SB diets.

**Table 4:** **Bone mineralization in growing female PCK rats fed different diets**

Measurement*	**Casein + CO**	**Casein + SO**	**SPI + SO**	**SPI + SB**	***P*** **-value**
**Femur**					
Ca (mg/g of bone)	93.0 ± 1.0	90.7 ± 1.7	94.3 ± 0.7	94.0 ± 1.5	0.23
P (mg/g of bone)	50.1 ± 0.5	49.0 ± 0.9	50.5 ± 0.4	50.5 ± 0.7	0.39
Ash (mg/g bone)	612.0 ± 7.3	578.9 ± 9.6	601.3 ± 8.2	614.5 ± 8.4	0.66
BMA (cm^2^)	1.30 ± 0.01	1.29 ± 0.02	1.28 ± 0.01	1.30 ± 0.01	0.27
BMC (g)	0.23 ± 0.0001	0.23 ± 0.0002	0.22 ± 0.0001	0.23 ± 0.0001	0.67
BMD (mg/cm^2^)	0.18 ± 0.0001	0.18 ± 0.0002	0.17 ± 0.0001	0.18 ± 0.0002	0.35
**Tibia**					
Ca (mg/g of bone)	90.7 ± 1.2	93.0 ± 1.1	96.8 ± 2.5	95.5 ± 1.9	0.14
P (mg/g of bone)	46.8 ± 0.9	48.3 ± 0.6	49.9 ± 1.3	49.5 ± 1.0	0.12
Ash (mg/g bone)	602.5 ± 5.9	592.1 ± 7.8	603.0 ± 7.5	606.7 ± 4.1	0.59
BMA (cm^2^)	1.54 ± 0.04	1.50 ± 0.06	1.56 ± 0.06	1.49 ± 0.04	0.56
BMC (g)	0.33 ± 0.01	0.32 ± 0.01	0.33 ± 0.01	0.31 ± 0.02	0.48
BMD (mg/cm^2^)	0.21 ± 0.002	0.21 ± 0.003	0.21 ± 0.001	0.21 ± 0.002	0.68

*Values are the means ± SEM of n = 12 rats/group. Abbreviations are *CO* corn oil, *SO* soybean oil, *SPI* soy protein isolate, *SB* 1:1 soybean oil:salmon oil blend, *BMA* bone mineral area, *BMC* bone mineral content, *BMD* bone mineral density, *Ca* calcium, *P* phosphorus.

### Bone microarchitecture

Shown in Table 5, there were significant differences in femur or tibia trabecular microarchitecture measurements of BV/TV, TbN, TbTH, TbSp, Conn or SMI in growing female PCK rats fed Casein + CO, Casein + SO, SPI + SO, and SPI + SB diets.

**Table 5:** **Trabecular bone microarchitecture in growing female PCK rats fed different diets**

Measurement*	**Casein + CO**	**Casein + SO**	**SPI + SO**	**SPI + SB**	***P*** **-value**
**Femur**					
BV/TV (%)	14.30 ± 0.54	14.00 ± 0.58	12.95 ± 0.96	13.64 ± 0.86	0.61
TbN (per mm)	2.87 ± 0.11	2.74 ± 0.11	2.77 ± 0.20	2.86 ± 0.10	0.87
TbTh (mm)	0.07 ± 0.0009	0.07 ± 0.001	0.06 ± 0.001	0.07 ± 0.002	0.18
TbSp (mm)	0.36 ± 0.01	0.38 ± 0.01	0.40 ± 0.04	0.36 ± 0.01	0.65
Conn (1/mm^3^)	80.77 ± 2.99	76.47 ± 2.83	77.06 ± 5.26	77.83 ± 3.59	0.86
SMI	2.28 ± 0.03	2.30 ± 0.05	2.12 ± 0.06	2.36 ± 0.07	0.31
**Tibia**					
BV/TV (%)	9.43 ± 0.62	9.67 ± 0.63	9.13 ± 0.84	9.24 ± 0.83	0.96
TbN (per mm)	2.51 ± 0.13	2.44 ± 0.17	2.39 ± 0.19	2.61 ± 0.16	0.82
TbTh (mm)	0.06 ± 0.001	0.06 ± 0.0009	0.06 ± 0.001	0.06 ± 0.001	0.33
TbSp (mm)	0.42 ± 0.02	0.44 ± 0.30	0.46 ± 0.05	0.41 ± 0.03	0.63
Conn (1/mm^3^)	43.00 ± 4.56	43.58 ± 4.59	43.53 ± 6.11	43.26 ± 6.34	1.00
SMI	2.85 ± 0.05	2.80 ± 0.04	2.88 ± 0.07	2.88 ± 0.07	0.72

*Values are expressed as the mean ± SEM of n = 12 bones/group. Abbreviations are *CO* corn oil, *SO* soybean oil, *SPI* soy protein isolate, *SB* 1:1 soybean oil:salmon oil blend, *BV/TV* trabecular bone volume per unit of total volume, *TbN* trabecular number, *TbTh* trabecular thickness, *TbSp* trabecular space, *Conn* connectivity, *SMI* structure model index.

There were also no significant differences in femur or tibia cortical thickness, cortical area, medullary area or porosity in growing female PCK rats fed different diets (Table 6).

**Table 6:** **Cortical bone microarchitecture in growing female PCK rats fed different diets**

Measurement*	**Casein + CO**	**Casein + SO**	**SPI + SO**	**SPI + SB**	**P value**
**Femur**					
Cortical Thickness (mm)	0.55 ± 0.008	0.55 ± 0.004	0.56 ± 0.004	0.55 ± 0.004	0.47
Cortical Area (mm^2^)	4.49 ± 0.03	4.40 ± 0.05	4.56 ± 0.03	4.52 ± 0.05	0.06
Medullary Area (mm^2^)	0.26 ± 0.006	0.25 ± 0.007	0.25 ± 0.006	0.26 ± 0.007	0.94
Porosity (%)	5.42 ± 0.12	5.45 ± 0.15	5.28 ± 0.14	5.39 ± 0.12	0.78
**Tibia**					
Cortical Thickness (mm)	0.52 ± 0.004	0.53 ± 0.003	0.53 ± 0.01	0.53 ± 0.006	0.59
Cortical Area (mm^2^)	2.90 ± 0.03	2.94 ± 0.03	2.88 ± 0.05	2.98 ± 0.04	0.29
Medullary Area (mm^2^)	0.20 ± 0.004	0.19 ± 0.003	0.18 ± 0.004	0.19 ± 0.005	0.46
Porosity (%)	6.32 ± 0.02	6.06 ± 0.09	6.11 ± 0.13	6.04 ± 0.16	0.37

*Values are expressed as the mean ± SEM of n = 12 bones/group. Abbreviations are *CO* corn oil, *SO* soybean oil, *SPI* soy protein isolate, *SB* 1:1 soybean oil:salmon oil.

### Bone biomechanical strength

There was no significant difference in femur or tibia biomechanical strength measures of peak force, ultimate stiffness, ultimate bending stress, and Young’s Modulus between the different diet groups (Table 7).

**Table 7:** **Bone strength measurements in growing female PCK rats fed different diets**

Measurement*	**Casein + CO**	**Casein + SO**	**SPI + SO**	**SPI + SB**	**P value**
**Femur**					
Peak Force (N)	37.2 ± 2.6	42.5 ± 2.7	40.4 ± 2.6	44.5 ± 3.0	0.26
Ultimate Stiffness (N/S)	437.6 ± 23.3	437.7 ± 37.1	385.6 ± 33.7	429.4 ± 29.8	0.63
Ultimate Bending Stress (N/mm^2^)	41.8 ± 3.2	47.3 ± 2.3	45.7 ± 3.0	48.6 ± 3.5	0.40
Young’s Modulus (N/mm^2^)	1160.6 ± 54.0	1171.5 ± 106.2	1040.0 ± 100.6	1117.8 ± 99.2	0.75
**Tibia**					
Peak Force (N)	19.2 ± 1.3	22.3 ± 1.7	20.0 ± 1.7	20.7 ± 1.3	0.57
Ultimate Stiffness (N/S)	178.0 ± 13.1	171.3 ± 13.0	158.9 ± 7.9	161.2 ± 10.9	0.61
Ultimate Bending Stress (N/mm^2^)	51.5 ± 3.4	61.7 ± 4.0	56.37 ± 5.58	57.41 ± 5.26	0.5
Young’s Modulus (N/mm^2^)	1938.8 ± 166.7	1970.0 ± 172.8	1815.6 ± 125.1	1765.5 ± 150.5	0.76

*Values are expressed as the mean ± SEM of n = 12 bone pairs/group. Abbreviations are *CO* corn oil, *SO* soybean oil, *SPI* soy prtein isolate, *SB* 1:1 soybean oil:salmon oil blend.

### Bone turnover markers

**Figure 2:**
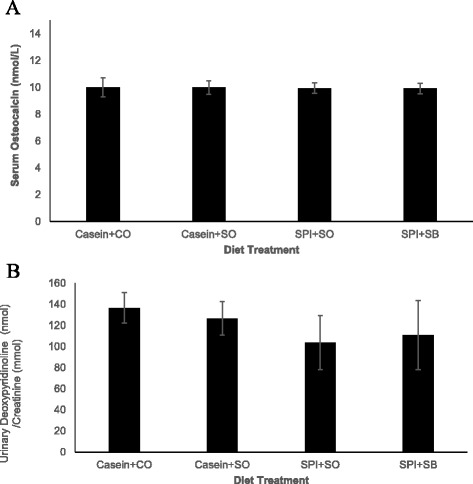
**The effect of feeding growing PCK female rats on soy protein isolate and/or omega-3 polyunsaturated fatty acids on body weight for 12 weeks on A) serum osteocalcin concentration and B) urinary deoxypyridinoline concentration.** Bars represent the mean ± SEM of n = 10 rats/diet group. Abbreviations are casein + corn oil (Casein + CO), casein + soybean oil (Casein + SO), soy protein isolate + soybean oil (SPI + SO) or soy protein isolate + 1:1 soybean/salmon oil (SPI + SB).

There were no significant differences in serum osteocalcin (Figure 2A) and urinary DPD concentration (Figure 2B) in growing female PCK rats fed the different experimental diets.

## Discussion

Hyperphosphatemia, hypocalcemia and hyperparathyroidism in children with chronic renal failure plays a significant role in bone disorders [1]. Liver disease can also contribute to bone disorders through impaired vitamin D synthesis which results in decreased Ca absorption and a compensatory increase in PTH production [23]. Despite greater (*P* = 0.01) urine output in the absence of higher water intake, urinary Ca excretion was significantly lower in rats fed SPI + SO diet compared to rats fed casein diets. Similarly, urinary P excretion was lower (*P* = 0.01) in SPI than casein-fed rats. Higher dietary Ca and P content in SPI diets resulted in greater Ca and P consumption. Lower urinary excretion and higher intake resulted in greater (*P* = 0.04) Ca and P retention in SPI-fed rats. PTH decreases Ca and P excretion in the urine; however, in the current study there were no statistical differences in serum PTH concentration among the diet groups. Failing kidneys lose capacity to concentrate urine. Plischke et al. [24] showed evidence linking fluid intake, vasopression, and osmotic control in PKD progression. In our study, reduced urinary Ca and P excretion in rats fed SPI diet may be due to lower urinary osmolality. Growing female PCK rats fed SPI + SO and SPI + SB diet had impaired renal function indicated by elevated serum blood urea nitrogen concentration compared to casein-fed rats [25]. Rats fed SPI + SB diet had significantly more renal cortical cyst obstruction [25] as well as significantly higher serum alkaline phosphatase and bilirubin concentrations due to hepatic cyst obstruction of the bile duct [26].

Despite higher Ca and P retention there were no differences on long bone Ca and P content in SPI compared to casein-fed rats. There were also no differences in femur or tibial BMA, BMC, and BMD as determined by DXA. In contrast, feeding growing female Sprague–Dawley rats SPI for 14 days enhanced tibial BMC and BMD compared to casein [10]. Differences may be due to a disease versus non-disease state. Also, metabolic abnormalities accompanying renal disease effects on bone microarchitecture cannot be detected by DXA [27]. Using μCT technology, we found no significant differences in femur and tibia trabecular microarchitecture in growing female PCK rats fed different diets. Lukas et al. [12] reported that growing female Sprague–Dawley rats fed oil sources rich in ALA increased femur and tibia BV/TV, TbN, TbTH and reduced TbSp by promoting bone formation indicated by higher serum osteocalcin concentration and no significant difference in bone resorption. In the present study, growing female PCK rats fed soybean oil or soybean oil: fish oil blend showed no significant differences in serum osteocalcin or urinary DPD concentration compared to rats fed corn oil. In our study, soybean oil was provided as a source of ALA, whereas in the Lukas et al. [12] study, flaxseed oil was used. The ALA content of flaxseed oil is higher 57% [20] than soybean oil (7.69%) or soybean:salmon oil blend (4.85%). Also, a higher dose (12% wt) of pure flaxseed oil or fish oil was provided to Sprague–Dawley rats compared to the oil dose (7% wt) provided to PCK rats. Therefore, higher n-3 PUFA doses may be necessary to enhance trabecular bone microarchitecture in growing female PCK rats. High fat diets were not fed to PCK rats due to potential to worsen polycystic kidney disease progression and severity [28]. Also, hypertension and cardiovascular defects are frequent complications of PKD that need to be considered when recommending a high fat diet [29-31]. In children with chronic kidney disease, cardiovascular disease is the most common cause of death and this has been suggested to be due in part to dysregulation of Ca and P homeostasis causing vascular calcification [32]. We did not assess vascular calcification, but found no significant differences in serum PTH, Ca or P concentration between diet groups.

In kidney disease, elevated PTH has been reported to preferentially cause cortical bone loss [27,33]. In the present study, no significant differences in femur and tibia cortical bone microarchitecture were observed in growing female PCK fed diets containing different protein and lipid sources. Growing female Sprague–Dawley rats fed oils rich in ALA, EPA, and/or DHA also showed no effect on cortical bone microarchitecture [12]. As expected in the absence of significant differences in bone mass and microarchitecture there were no significant differences in bone strength measurements between growing female PCK rats fed different diets.

Besides mineral imbalance and failure to achieve PBM, children with kidney disease also suffer growth disturbances [34]. Early management is required to prevent growth failure and shorter stature [2]. In the present study, all diets supported growth indicated by no significant differences in body weights. A long-term (two years) study showed no difference in body weight gain in Sprague–Dawley rats fed a soy protein compared to a casein-based diet [35]. Similar body weight gain has also been reported for growing rats fed soybean oil, flaxseed oil, and fish oil [20,36]. Hans:SPRD-*cy* rats provided soy protein *ad libitum* had higher body weights and showed improved kidney cyst volume and renal function compared to rats fed casein [37]. In our study female PCK rats fed SPI diets showed higher final urinary output and decreased renal function compared to casein-fed rats; however, there were no significant body weight differences. This may have been because all diets were restricted to 15 g/d.

In our study, femur length was shorter in rats fed SPI + SO diet compared to rats fed casein diets. SPI contains isoflavone phytoestrogens with binding specificity for estrogen receptor beta [38]. Estrogen receptor beta has been shown to be a physiological inhibitor of bone growth [39]. Tou et al. [40] reported that female Sprague–Dawley rats fed a phytoestrogen diet shortened (*P* < 0.05) femur length compared to rats fed a casein diet. However, PCK rats fed SPI + SB diet had no effect on femur length. The type of lipid associated with SPI may influence bone. Of the diet groups, SPI + SB diet consisting of a soybean oil:fish oil blend had the highest n-3:n-6 PUFA ratio as well as EPA and DHA content. Growing female Hans:SPRD-*cy* rats, a model of ADPKD, fed casein with 7% wt ALA-rich flaxseed oil for 12 weeks resulted in longer femurs compared to rats fed corn oil low in ALA [41]. ALA can be converted to EPA and DHA *de novo* [42]. Although conversion is inefficient, higher amounts of ALA associated with flaxseed oil can result in higher EPA and DHA content. Mechanisms whereby n-3 PUFAs have been suggested to promote bone growth include reducing prostaglandin E_2_ production by decreasing the n-6 to n-3 PUFA ratio and by increasing circulating insulin-like growth factor binding protein to promote growth factors [43,44]. Estrogen can simulate the conversion of ALA into their longer-chain metabolites [45]. Therefore, a potential synergistic effect of SPI and n-3 PUFAs is estrogenic effects of isoflavones associated with SPI may increase conversion of ALA to long-chain n-3 PUFAs. Female PCK rats fed soybean oil with protein source provided as either SPI or casein showed no significant differences in serum and liver EPA, DHA, and ARA content [25,26]. However, dietary groups in our study did not allow us to specifically assess synergistic effects of protein and n-3 PUFAs on femur longitudinal growth.

Another limitation of this study was the AIN-93G diet is based on a purified casein diet. This standard purified diet does not meet the NRC recommendations for sulfur amino acids to support rodent growth and requires the addition of L-cysteine [46]. Therefore, SPI diets were supplemented with L-cysteine to equal casein diets. This potentially alters acid load typically associated with the amino acid profiles of these proteins. Also, a longer study duration than 12 weeks may be required to detect dietary effects of enhance bone mineralization due to higher Ca and P retention. However, ARPKD onset occurs early in childhood with renal failure frequently occurring in the first decade of life [6]. Although, the PCK rat model has the advantage of being a slow progressing form of ARPKD, the study was ended at 12 weeks due to morbidity associated with severity of polycystic liver [26,47].

## Conclusion

In summary, to our knowledge this was the first study to investigate diet as a therapeutic strategy aimed at bone health in PCK rats, an orthologous model of human ARPKD [8,47]. Current general dietary guidelines to promote a healthier population include increasing consumption of plant proteins and n-3 PUFAs [48]. Growing female PCK rats fed SPI diets increased Ca and P retention, but not bone Ca and P content. All diets equally supported body weight gain and produced no greater or worse long bone mass, microarchitecture, and strength, but femur longitudinal growth was affected. Based on the results dietary SPI and n-3 PUFA supplementation influenced mineral balance without enhancing bone health in ARPKD.

## Abbreviations

ADPKD: Autosomal recessive polycystic kidney disease
AIN-93G: American Institute of Nutrition-93G
ALA: Alpha-linolenic acid
ARPKD: Autosomal recessive polycystic kidney disease
BMA: Bone mineral area
BMC: Bone mineral content
BMD: Bone mineral density
BV/TV: Bone volume per unit of total volume
Ca: Calcium
ddH_2_O: Deionized distilled water
DPD: Deoxypyridinoline
DHA: Docosahexaeonic acid
DXA: Dual energy X-ray absorptiometry
EIA: Enzyme immunoassay
EPA: Eicosapentaenoic acid
ICP: Inductively coupled plasma spectrometry
LA: Linoleic acid
n-3 PUFA: Omega-3 polyunsaturated fatty acids
n-6 PUFA: Omega-6 polyunsaturated fatty acids
μCT: Micro-computed tomography
PBM: Peak bone mass
P: Phosphorus
SB: Soybean oil:salmon oil blend
SEM: Standard error of the mean
SPI: Soy protein isolate
SMI: Structure model index
SO: Soybean oil
TbN: Trabecular number
TbTH: Trabecular thickness
TbSp: Trabecular separation
